# Circulating MicroRNAs as Biomarkers for the Early Diagnosis of Lung Cancer and Its Differentiation from Tuberculosis

**DOI:** 10.3390/diagnostics14232684

**Published:** 2024-11-28

**Authors:** Yeldar Ashirbekov, Nazgul Khamitova, Kantemir Satken, Arman Abaildayev, Ilya Pinskiy, Askar Yeleussizov, Laura Yegenova, Anargul Kairanbayeva, Danara Kadirshe, Gulzhakhan Utegenova, Nurlan Jainakbayev, Kamalidin Sharipov

**Affiliations:** 1 Aitkhozhin Institute of Molecular Biology and Biochemistry, Almaty 050012, Kazakhstan; eldarasher@mail.ru (Y.A.); nazgul2608@mail.ru (N.K.); hdeathless@gmail.com (K.S.); armandj_92@mail.ru (A.A.); dkadirse@gmail.com (D.K.); skamalidin@mail.ru (K.S.); 2 Al-Farabi Kazakh National University, Almaty 050040, Kazakhstan; ilya.pinskyi@mail.ru; 3 Kazakh National Medical University Named After S.D. Asfendiyarov, Almaty 050012, Kazakhstan; 4 Kazakh Institute of Oncology and Radiology, Almaty 050012, Kazakhstan; askar.m.yeleussizov@gmail.com; 5 National Scientific Center of Phthisiopulmonology, Almaty 050010, Kazakhstan; egenova@mail.ru (L.Y.); anarka_19_91@mail.ru (A.K.); 6 South Kazakhstan Pedagogical University Named After Ozbekali Zhanibekov, Shymkent 160012, Kazakhstan; 7 Kazakh-Russian Medical University, Almaty 050004, Kazakhstan; tnd1101@gmail.com

**Keywords:** lung cancer, tuberculosis, differential diagnosis, microRNA

## Abstract

Background: The differential diagnosis of tuberculosis (TB) and lung cancer (LC) is often challenging due to similar clinicopathological presentations when bacterial shedding is negative, which can lead to delays in treatment. In this study, we tested the potential of plasma-circulating microRNAs (miRNAs) for the early and differential diagnosis of TB and LC. Methods: We conducted a two-phase study: profiling 188 miRNAs in pooled plasma samples and validating 14 selected miRNAs in individual plasma samples from 68 LC patients, 38 pulmonary TB patients, and 41 healthy controls. Results: Twelve miRNAs were significantly elevated in LC patients compared to controls and TB patients, while two miRNAs were significantly elevated in TB patients compared to controls. ROC analysis demonstrated that miR-130b-3p, miR-1-3p, miR-423-5p, and miR-200a-3p had good discriminatory ability to distinguish LC patients (including those with stage I tumours) from healthy individuals and miR-130b-3p, miR-423-5p, miR-15b-5p, and miR-18b-5p effectively distinguished LC patients (including those with stage I tumours) from TB patients. Additionally, miR-18b-5p showed good discriminatory ability between SCLC and NSCLC patients. Conclusions: Circulating miRNAs hold strong potential for the early detection of LC and for distinguishing LC from TB.

## 1. Introduction

Tuberculosis (TB) and lung cancer (LC) are among the most pressing medical and social challenges worldwide. These issues arise from the widespread prevalence of these diseases, high mortality rates, and patient disability.

TB is an infection caused by the aerobic non-motile bacterium *Mycobacterium tuberculosis* (*Mtb*) which primarily affects the lungs. The WHO estimates that in 2022, 10.6 million people worldwide fell ill with TB, and 1.3 million died. Before the emergence of COVID-19, TB was the leading cause of death from a single infectious agent [1]. TB diagnosis involves verification in patients with clinical and radiological signs, but negative results from diagnostic tests (microscopy, culture, PCR tests) do not rule out closed TB; they only indicate a lack of bacterial excretion [2]. Patients without a confirmed diagnosis undergoing anti-TB treatment must accept the risks of toxic side effects from medications (primarily liver damage).

LC is one of the most common cancers and the leading cause of death from malignant neoplasms. In 2022, 2.5 million new cases of LC were registered globally, with 1.8 million deaths [3]. This high mortality rate is largely due to late detection, as more than 70% of tumours are diagnosed at stages III and IV when therapeutic options are limited. Consequently, the average five-year survival rate after diagnosis of LC is only 10–20% in most countries [4]. The main reasons for late detection are the asymptomatic course of the disease in its early stages, nonspecific symptoms, and rapid tumour progression. LC diagnosis requires a biopsy of the suspected tissue and histological examination for cancer cells, but the existing methods for collecting biomaterial are invasive and not always feasible [5].

For both diseases, timely diagnosis is critical for successful treatment. However, due to the high prevalence of TB and the similar clinical and radiological presentation of diseases, many LC patients initially receive anti-TB treatment, resulting in diagnostic delays, tumour progression, and drug toxicity [6]. Conversely, TB can be misdiagnosed as LC [6,7,8]. Cases of concomitant TB and LC are particularly problematic due to delayed diagnosis of one of the diseases and therapeutic limitations [7,9].

These challenges in early and differential diagnosis of TB and LC have led to the search for new minimally invasive methods. MicroRNAs (miRNAs) are being explored as promising biomarkers for disease diagnosis. miRNAs are small single-stranded, non-coding RNAs that function as negative regulators of genes. Their biological significance, biogenesis, and mechanisms of action have been well described in previous works [10,11].

miRNAs play an important role in *Mtb* evasion of the host’s immune system by influencing macrophage metabolism, suppressing inflammatory responses, and regulating apoptosis and autophagy, allowing the infection to persist in the body [12]. Studies have demonstrated the potential of circulating miRNAs for diagnosing active TB [13,14,15,16,17,18], differentiating TB from chronic obstructive pulmonary disease (COPD), pneumonia, and LC [13], distinguishing between latent and active forms of TB [14,15], identifying drug-resistant TB [19], and assessing treatment response [15,20,21].

The role of miRNAs in cancer development is well-established. miRNAs are involved in the onset, progression, and metastasis of malignant tumours and are, therefore, considered as promising cancer biomarkers [22,23]. They enter the bloodstream directly from primary or metastatic tumours via active secretion, apoptosis, or necrosis, so changes in circulating miRNA levels can reflect the underlying pathological process [24]. Many miRNAs have been recommended as plasma/serum diagnostic and prognostic markers for LC [25,26,27,28,29,30,31,32,33,34]. A significant issue with such studies is the low reproducibility of results across different investigations, possibly due to population differences, lifestyle factors, and variations in collection, extraction, and quantification methodologies. Some studies indicate that the pathogenesis of cancer may have ethnic-specific features [35,36], which also applies to the applicability of circulating miRNAs as markers [37,38].

Our study aimed to evaluate the potential of circulating plasma miRNAs as biomarkers for the differential diagnosis of TB and LC in the population of Kazakhstan. We conducted a comparative quantitative analysis of plasma miRNA levels among three groups: LC patients (*N* = 64), TB patients (*N* = 35), and healthy controls (*N* = 39). In the first exploratory phase, we tested 188 candidate miRNAs on five pooled plasma samples and selected 14 promising candidates. In the second phase, we validated them using a total sample of individual plasma specimens.

## 2. Materials and Methods

### 2.1. Ethical Statement

The study was approved by the local ethics committee of the Aitkhozhin Institute of Molecular Biology and Biochemistry. Informed consent was obtained from all subjects whose biomaterial was used in the study.

### 2.2. Subjects

Venous blood sampling for the study was conducted at three medical institutions. Blood samples from 82 primary patients with histologically confirmed LC (LC patients) and 1 patient with laboratory-confirmed TB were collected at the Kazakh Research Institute of Oncology and Radiology, Almaty, Kazakhstan, before the start of therapy, from July 2022 to June 2023. Blood samples from 40 patients with laboratory-confirmed TB (TB patients) were collected at the National Scientific Center of Phthisiopulmonology, Almaty, Kazakhstan, before the start of therapy, from September to December 2022. Inclusion criteria for all patients: adults, confirmed diagnosis, prior to therapy. Exclusion criteria: severe comorbidities, HIV, other cancers, mental diseases. Blood samples from 41 healthy donors (Controls) were collected at the Almaty Multidisciplinary Clinical Hospital between January and March 2023. Blood was collected in vacuum tubes containing 3.2% sodium citrate as an anticoagulant, which demonstrated a considerable miRNA yield during subsequent RNA extraction in preliminary tests.

### 2.3. Plasma Preparation and RNA Isolation

Before plasma extraction, the blood was stored for no more than 2 h at 4 °C. After gentle mixing, the blood samples were centrifuged at 1000× *g* for 15 min at 4 °C. The supernatant was then transferred to a new tube and subjected to a second centrifugation at 2500× *g* for 15 min at 4 °C. The resultant plasma was divided into 100 μL aliquots and stored at −70 °C until further use.

Total RNA was isolated from 100 μL of plasma using a commercial MagMAX mirVana Total RNA Isolation Kit (ThermoFisher A27828, Waltham, MA, USA) according to the manufacturer’s protocol. Before RNA isolation, 2 fmol of the exogenous spike-in control ath-miR-159a (ordered as a 5′-phosphorylated RNA oligonucleotide, ThermoFisher 10620310) was added to each sample.

### 2.4. Obtaining cDNA and Quantitative PCR

Immediately after RNA isolation, cDNA synthesis and quantitative PCR reactions were performed. cDNA was synthesized using the TaqMan Advanced miRNA cDNA Synthesis Kit (ThermoFisher A28007) according to the manufacturer’s protocol. Quantitative PCR was carried out using specific primers and hydrolysis probes from the TaqMan Advanced miRNA Assays kit (ThermoFisher A25576) and the TaqMan Fast Advanced Master Mix reagent (ThermoFisher 4444556, 4444557, 4444964) under the conditions recommended by the manufacturer on the StepOnePlus Real-Time PCR System (ThermoFisher 4376600, discontinued).

Primary data processing was performed using StepOnePlus 2.2.2 and ExpressionSuite v1.3 software. Relative quantification was conducted using the comparative Ct (ΔΔCt) method with modifications described by Königshoff et al., 2009 [39]. Relative transcript abundance is expressed in ΔCt values (ΔCt = Ct_reference_ − Ct_target_). The exogenous spike-in control ath-miR-159a was used as the reference. The ΔΔCt value (ΔΔCt = mean ΔCt_case_ − mean ΔCt_control_) was interpreted as log_2_ fold change (log_2_FC).

### 2.5. Study Design

The study consisted of two stages: exploratory miRNA profiling and validation.

At the first exploratory stage, using TaqMan Advanced miRNA Human Serum/Plasma 96-well Plates (ThermoFisher A31878, Waltham, MA USA), we tested 188 miRNAs on five pooled plasma samples from the following groups: 10 patients with adenocarcinoma (AC), 10 patients with squamous cell carcinoma (SCC), 9 patients with small cell carcinoma (SCLC), 10 TB patients, and 10 healthy controls. The selection of promising candidate miRNAs was carried out based on three parameters: (1) amplification quality (amplification score > 0.6); (2) the magnitude of fold changes between groups (FC > 3); (3) the required classifier criteria (to discriminate between LC patients and healthy individuals, TB patients and healthy individuals, LC patients and TB patients, and between patients with different histological types of LC). The amplification score was derived from the ExpressionSuite v1.3 software. Heatmap diagrams showing comparative miRNA expressions between groups were generated using the SRplot online platform [40]. Venn diagrams showing the overlap of the differentially expressed miRNAs among groups were created using the online tool [41].

To validate the results of the miRNA profiling, the selected miRNAs were tested on individual plasma samples from 68 LC patients, 38 TB patients, and 41 healthy controls. Most of the samples from the exploratory stage were randomly included in the validation set, including 4 from AC patients, 4 from SCC patients, 8 from SCLC patients, 7 from TB patients, and 10 from healthy controls. For comparative visualization of miRNA levels between groups, boxplots were generated using the SRplot online platform [40].

### 2.6. Statistical Analysis

Most statistical calculations were performed using Jamovi v2.2.5.0 software [42]. To compare the characteristics of the studied groups, we applied the Mann–Whitney *U* test for quantitative data and Pearson’s Chi-squared test (χ^2^-test) for nominal data. The statistical significance of differences in miRNA levels between the groups was assessed using the Mann–Whitney *U* test. *p*-values < 0.05 were considered statistically significant. For multiple comparisons, *p*-values were adjusted using the False Discovery Rate (FDR) online calculator [43]. The characteristics of the markers were evaluated based on receiver operating characteristic (ROC) analysis, calculated using the Web tool for ROC analysis [44] and the online ROC calculator [45]. The optimal cut-off point was determined using Youden’s index method. Evaluation of classifiers by interpretation of the area under the ROC curve (AUC) was conducted as described by Muller et al., 2005 [46].

## 3. Results

### 3.1. Characteristics of the Compared Groups

The characteristics of the compared groups are presented in Table 1. In all the studied groups, the vast majority of individuals were Kazakhs, with other ethnic groups including Uyghurs, Russians, Tatars, Mari, and Dungans.

The exploratory set consisted of five groups: 10 AC patients, 10 SCC patients, 9 SCLC patients, 10 TB patients, and 10 healthy controls. The TB patients and controls did not differ in age, gender distribution, or number of smokers. The AC, SCC, and SCLC patients did not differ significantly from each other but differed significantly in age from the TB patients (*p* = 0.029, 0.012, and 0.035, respectively) and from the controls (*p* = 0.002, 9.9 × 10^−4^, and 0.010, respectively). Males predominated in all groups. The proportion of smokers was significantly higher among the SCC patients compared to AC patients, SCLC patients, TB patients, and controls (*p* = 0.003, 0.047, 0.003, and 0.010, respectively).

The validation set included 68 LC patients, 38 TB patients, and 41 healthy controls. The LC patients and controls did not differ significantly in age, gender distribution, or the number of smokers; however, both groups differed significantly from the TB patients in these parameters. On average, the TB patients were 20 years younger than the other two groups (*p* = 1.5 × 10^−8^ and 3.9 × 10^−7^, respectively). The gender ratio among TB patients was approximately equal, whereas there were significantly more males in the LC and control groups (*p* = 0.014 and 0.010, respectively). There were significantly fewer smokers among the TB patients compared to the controls, and especially compared to the LC patients (*p* = 0.012 and 1.4 × 10^−4^, respectively). The proportion of patients with drug-resistant TB was 26% of all TB patients, consistent with official statistical data for the Republic of Kazakhstan. Most LC patients (62%) had tumours at advanced stages (III or IV), which aligns with the literature data indicating predominantly late detection of LC. The tumour-type distribution roughly matched the expected frequencies.

### 3.2. miRNA Profiling

**Figure 1:**
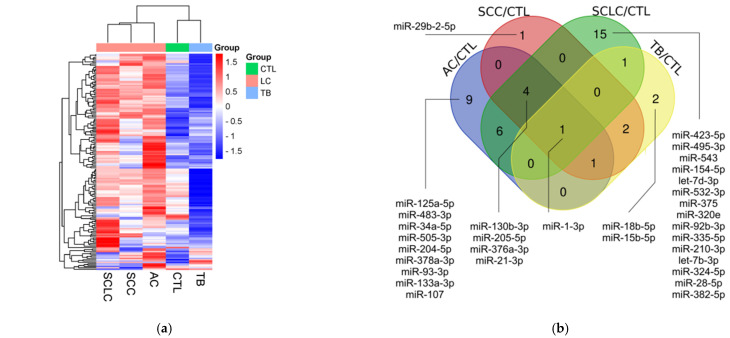

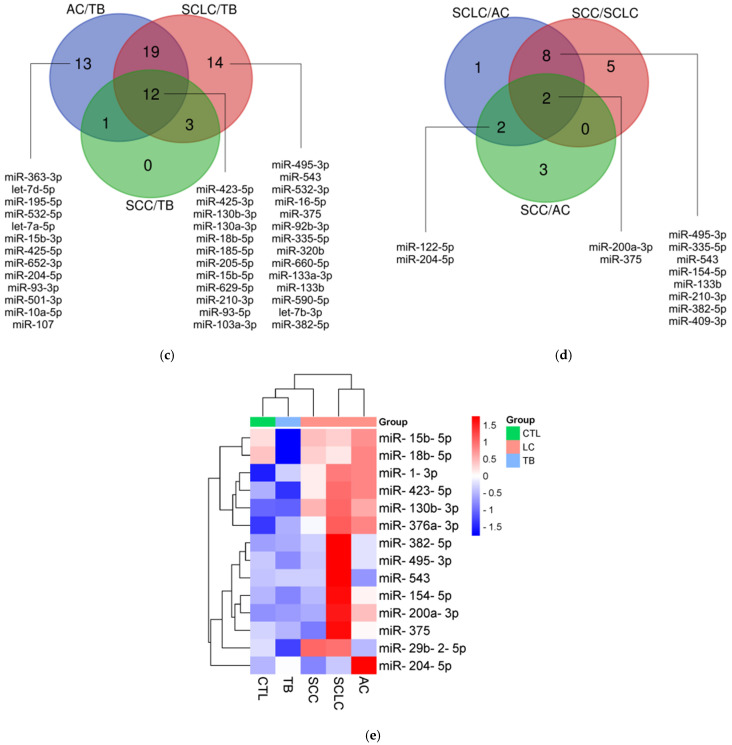
miRNA profiling results: (**a**) heatmap diagram of the levels of 161 out of 188 miRNAs remaining after the first selection based on amplification quality; (**b**) Venn diagram illustrating the distribution of miRNAs with notably altered level (FC > 3) in four groups of patients compared to the control group; (**c**) Venn diagram illustrating the distribution of miRNAs with notably altered level (FC > 3) in three groups of LC patients compared to TB patients; (**d**) Venn diagram illustrating the distribution of miRNAs with notably altered level (FC > 3) among the three groups of LC patients compared with each other; (**e**) heatmap diagram of the levels of 14 miRNAs remaining after the final selection.

The miRNA profiling results are shown in Figure 1. Briefly, we compared the levels of 188 miRNAs in 5 pooled samples from 10 AC patients, 10 SCC patients, 9 SCLC patients, 10 TB patients, and 10 healthy controls. First, we filtered out all miRNAs without amplification or with Ct above the threshold of 35. Second, we excluded miRNAs with low amplification quality, using an Amplification score of 0.6 as the threshold. The remaining 161 miRNAs were included in the selection process (Figure 1a). The groups were compared, and miRNAs with a difference of 3-fold or more between the groups were selected. From these 70 miRNAs, those potentially useful for diagnosing LC and TB (Figure 1b), for the differential diagnosis of LC and TB (Figure 1c), as well as for the differential diagnosis of SCLC and NSCLC (Figure 1d), were chosen. In the end, 14 miRNAs were selected (Figure 1e). The list could have been broader, but the number of available slots was limited.

### 3.3. Validation

**Figure 2:**
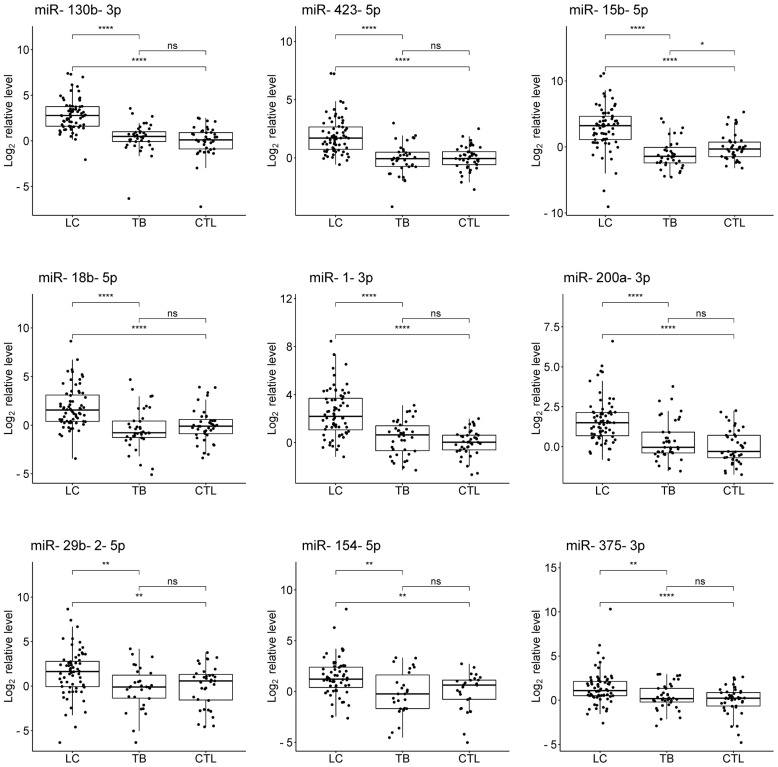

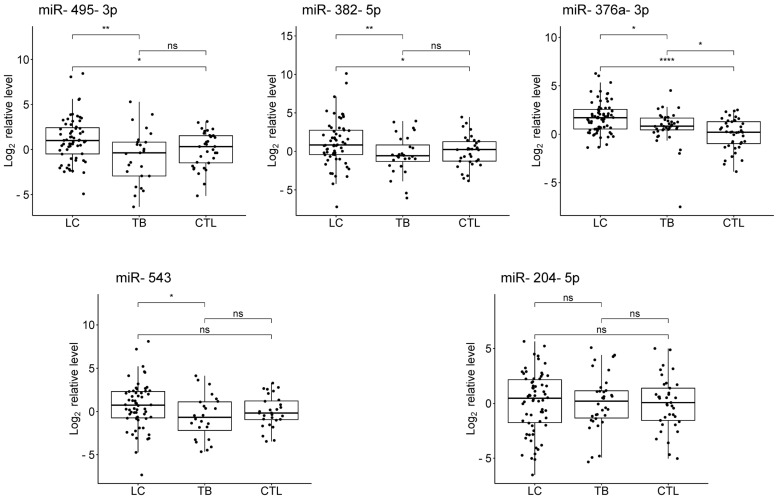
Plasma levels of 14 miRNAs in the validation set of 68 LC patients, 38 TB patients, and 41 healthy controls. The statistical significance was assessed using the Mann-Whitney *U* test: ns—not significant; * *p* < 0.05; ** *p* < 0.01; **** *p* < 0.0001.

The results of the exploratory stage were validated on individual samples from 68 LC patients, 38 TB patients, 4 patients with suspected LC, 9 patients with suspected TB, and 41 healthy controls (Supplementary Table S1). Comparative quantitative statistics between the three groups are presented in Table 2. A comparison of the levels of the studied miRNAs between the groups is shown in Figure 2.

The levels of miR-130b-3p, miR-1-3p, miR-423-5p, miR-200a-3p, miR-15b-5p, miR-18b-5p, miR-376a-3p, miR-375-3p, miR-154-5p, miR-29b-2-5p, miR-382-5p, and miR-495-3p were significantly elevated in the plasma of LC patients compared to controls, with log_2_FC ranging from 0.9 for miR-495-3p to 3.2 for miR-15b-5p. Ten out of the twelve significant differences remained significant after adjustment for multiple testing. The levels of miR-543 and miR-204-5p showed no significant changes in LC patients compared to controls.

The level of miR-376a-3p was significantly elevated, and the level of miR-15b-5p was significantly reduced in TB patients compared to controls; however, log_2_FC was small in both cases. Both differences remained significant after adjustment for multiple tests. The levels of the remaining 12 miRNAs showed no significant changes in TB patients compared to controls.

The levels of miR-130b-3p, miR-423-5p, miR-15b-5p, miR-18b-5p, miR-1-3p, miR-200a-3p, miR-29b-2-5p, miR-154-5p, miR-375-3p, miR-495-3p, miR-382-5p, miR-376a-3p, and miR-543 were significantly elevated in LC patients compared to TB patients, with log_2_FC ranging from 0.76 for miR-376a-3p to 4.31 for miR-15b-5p. Twelve out of the thirteen significant differences remained significant after adjustment for multiple testing. The level of miR-204-5p showed no significant changes between LC and TB patients.

To evaluate whether the inclusion of samples from the exploratory stage in the validation set affected the results, we compared the groups after excluding these samples. The analysis conducted on a fully independent validation sample set confirmed that all identified changes remained consistent (Supplementary Table S2).

Considering the potential use of miRNA markers in the early diagnosis of LC, we examined the levels of our miRNAs in stage I LC patients. All miRNAs that were significantly elevated in the overall group of LC patients were also significantly elevated in stage I LC patients when compared to TB patients and healthy controls (Supplementary Table S3).

In addition, 22 significant differences in miRNA levels were identified between groups with different clinicopathological parameters; however, after adjustment for multiple testing, only three of them remained significant (Supplementary Table S4). The levels of miR-200a-3p and miR-375-3p were significantly elevated in AC patients compared to SCC patients. The levels of miR-18b-5p, miR-154-5p, miR-375-3p, and miR-29b-2-5p were significantly elevated in SCLC patients compared to AC patients. The levels of miR-18b-5p, miR-375-3p, miR-154-5p, miR-130b-3p, miR-200a-3p, and miR-495-3p were significantly elevated in SCLC patients compared to SCC patients. The levels of miR-18b-5p, miR-375-3p, miR-154-5p, miR-130b-3p, miR-29b-2-5p, miR-495-3p, and miR-15b-5p were significantly elevated in SCLC patients compared to NCLC patients. The level of miR-375-3p was significantly elevated in LC patients with metastasis compared to those without metastasis. The level of miR-204-5p was significantly elevated in TB patients who are smokers compared to non-smokers. The level of miR-423-5p was significantly elevated in healthy smokers compared to healthy non-smokers. There were no significant differences in miRNA levels between genders in the three groups or between patients with drug-sensitive and drug-resistant TB.

To assess the impact of age on differences in miRNA levels between groups, we conducted regression analysis and partial correlation analysis. Both methods indicated that age did not affect the observed differences in the levels of most miRNAs (Supplementary Table S5).

### 3.4. Evaluation of the Diagnostic Potential of miRNAs

To assess the diagnostic abilities of miRNAs that showed significant differences in levels between groups, we conducted ROC analysis. The evaluation of the discriminative ability of potential markers by interpretation of AUC was conducted as described by Muller et al., 2005 [46]: excellent discrimination, AUC of ≥0.90; good discrimination, 0.80 ≤ AUC < 0.90; fair discrimination, 0.70 ≤ AUC < 0.80; and poor discrimination, AUC of <0.70.

**Figure 3:**
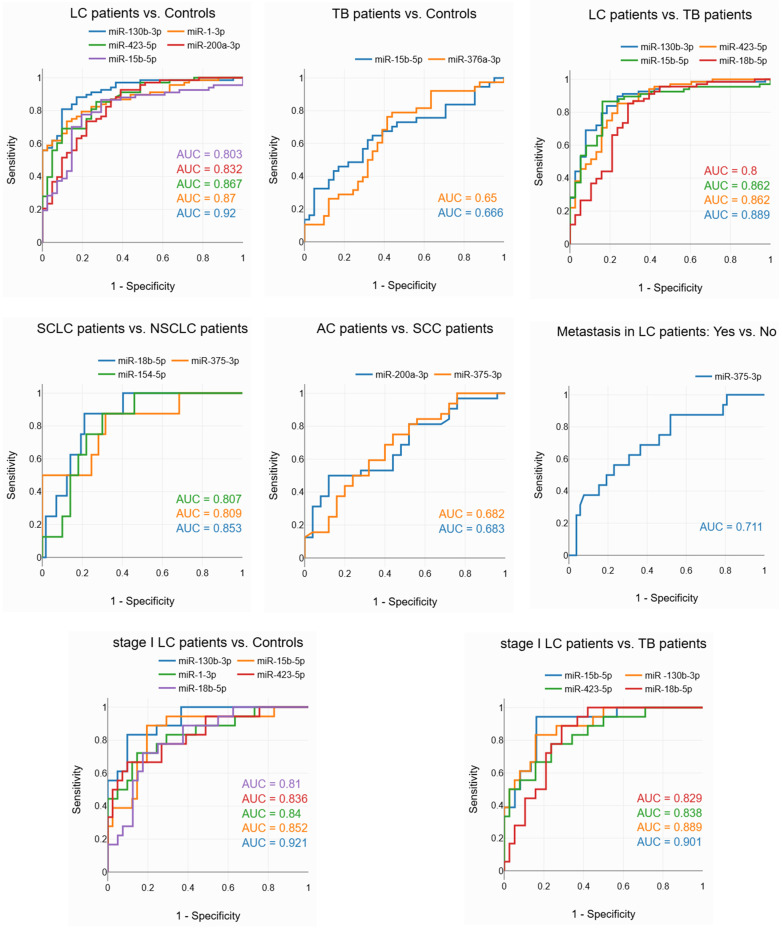
ROC curves showing the ability of miRNAs to discriminate between studied groups.

We tested six potential applications of the miRNA marker. The results of the ROC analysis are presented in Supplementary Table S6. The analysis showed that miRNAs have a good ability to discriminate between LC patients and healthy individuals, between LC and TB patients, and between SCLC and non-small cell LC (NSCLC) patients; a fair ability to discriminate between LC patients with and without metastasis; and a poor ability to discriminate between TB patients and healthy individuals, and between AC and SCC patients (Figure 3).

Furthermore, miRNAs exhibit good ability to discriminate between stage I LC patients and healthy controls, as well as between stage I LC patients and TB patients. These results suggest that miRNAs have potential for early LC diagnosis and for differential diagnosis between early LC and TB.

## 4. Discussion

TB and LC remain significant health issues worldwide, particularly in Kazakhstan. The challenges in diagnosing these and other pulmonary diseases with similar clinical and radiological presentations underscore the need for new minimally invasive methods for early and differential diagnosis. In our study, we evaluated the potential of plasma-circulating miRNAs as biomarkers for the early and differential diagnosis of TB and LC in the Kazakhstani population. To achieve this, we examined and validated the plasma miRNA profiles of LC and TB patients in comparison with each other and with healthy controls. Our findings indicate that plasma miRNAs hold a high potential for diagnosing LC (including early detection) and for distinguishing between LC and TB. Contrary to previous reports proposing miRNAs as biomarkers for TB [13,14,15,16,17,18,19,20,21], our study did not find significant miRNAs with diagnostic utility specifically for TB.

The greatest potential for the differential diagnosis of LC and TB was shown by miR-130b-3p and miR-423-5p. A literature search indicates that these two miRNAs play a significant role in the pathogenesis of LC. Chen et al. [47] showed that miR-130b-3p promotes immune escape and metastasis in NSCLC by down-regulating the tumour suppressor STK11. Guo et al. [48] demonstrated that miR-130b-3p is overexpressed in NSCLC and that miR-130b-3p, conveyed through mesenchymal stem cell-derived extracellular vesicles, advances LC progression by inhibiting the FOXO3/NFE2L2/TXNRD1 axis. Studies have shown that increased miR-130b-3p expression enhances angiogenesis and tumour progression in various cancers [49,50,51]. Elevated miR-130b-3p expression has been observed in oral cancer [50], gastric cancer [52], liver cancer [49], colorectal cancer [53], bladder cancer [54], and prostate cancer [55]. These studies align with our findings, reinforcing the role of miR-130b-3p as a relevant biomarker and a potential therapeutic target.

In contrast to our findings and the studies mentioned above, Lv et al. [56] report a protective role of miR-130b-3p in NSCLC. They found that miR-130b-3p inhibits DEPDC1 expression, which, via the TGF-β signalling pathway, leads to reduced proliferation, migration, and apoptosis suppression in cancer cells; additionally, a decrease in serum exosomal miR-130b-3p was noted in NSCLC patients. In our study, we observed the opposite effect—a more than six-fold increase in plasma miR-130b-3p levels compared to the control group.

There are fewer studies on miR-423-5p, but sufficient evidence suggests its significant role in carcinogenesis. Huang et al. [57] identified it as a promoter of lung AC through inhibition of CADM1, facilitating cell proliferation and invasion. Wang et al. [58] demonstrated that miR-423-5p accelerates NSCLC progression by targeting SLIT2. Sun et al. [59] found that elevated miR-423-5p expression in lung AC contributes to brain metastasis by directly inhibiting MTSS1. In contrast to these studies, which support our findings, the work of Yu et al. [60] and Li et al. [61] reported a protective role of miR-423-5p in LC through inhibition of the oncogenes CEACAM1 and MYBL2.

Particularly relevant to our study is the work by Kang et al. [62], which examines both miR-130b-3p and miR-423-5p in the context of TB and LC interaction. The authors demonstrated that TB promotes the proliferation of cancer cells through the influence of exosomal miR-130b-3p and miR-423-5p, which enhance the expression of Cyclin D1 and p65. The study reports a high concentration of these miRNAs in exosomes from pleural effusion; however, blood plasma was not examined. In another study, Tu et al. [16] reported increased serum levels of miR-423-5p in TB patients. The authors found that miR-423-5p is linked to TB through its inhibitory effect on autophagosome-lysosome fusion in macrophages—a crucial immune defence process—by directly targeting VPS33A, which may facilitate TB infection. According to our data, the concentration of miR-130b-3p and miR-423-5p in the blood plasma of TB patients is not elevated, in contrast to LC patients, which enables differentiation between the two conditions.

In addition to miR-130b-3p and miR-423-5p, our study identified other miRNAs with valuable diagnostic applications. Compared to these two, miR-15b-5p demonstrated lower specificity for distinguishing LC from healthy controls, while miR-1-3p was less specific for differentiating LC from TB. MiR-18b-5p displayed strong discriminatory power in differentiating SCLC from NSCLC, which is important for selecting appropriate treatment strategies.

Notably, the miRNAs we propose are valuable for early and differential diagnosis of early LC, as they retained strong discriminatory power when comparing only stage I LC patients to healthy controls and TB patients.

Several studies have explored miRNAs as differential diagnostic markers for some lung diseases, but such research remains sparse. Previously, miRNA biomarkers were investigated for distinguishing TB from pneumonia, LC and COPD [13], LC from COPD [25,63], LC from bronchiectasis [25], LC from pneumonia [25,64], and LC from benign lung lesions [34]. To our knowledge, only a single study by Zhang et al. [13] examined miRNAs specifically for differentiating TB from LC. The scarcity of such studies emphasizes the importance of our findings, which add valuable data to the limited research on miRNA-based differential diagnostics in pulmonary diseases.

The commercial kit we used in the exploratory phase, consisting of two 96-well plates, is specifically optimized for profiling 188 miRNAs in blood serum/plasma. We suggest that this design allowed for broad coverage of circulating miRNAs, facilitating the identification of relevant biomarkers.

Kim et al. [65] demonstrated that the choice of anticoagulant can significantly affect miRNA yield, which is important given their low abundance in plasma. In line with their findings, our preliminary tests showed that, among anticoagulants, the best yield was obtained with a mixture of sodium fluoride and potassium oxalate, followed closely by sodium citrate. Given its availability, we used sodium citrate (3.2%) and recommend it over EDTA for similar studies.

Although our findings are supported by the existing literature, reproducibility in miRNA studies remains a challenge. This variability may arise from biological factors, such as inter-population differences, or from methodological inconsistencies that are often unreported. Standardizing protocols could address many of these discrepancies. However, population-specific factors, including genetic background and environmental exposures, likely contribute to variability in miRNA expression, underscoring the need for biomarker validation in specific populations.

Further studies are necessary to confirm the clinical applicability of our results. Future research should expand the sample size, increase the range of different pulmonary diseases and, where appropriate, explore additional confounding variables. Additionally, longitudinal studies could assess the predictive value of these biomarkers for disease progression, treatment response, and prognosis, thereby contributing to personalized treatment strategies.

## 5. Conclusions

In summary, our study shows that while circulating miRNAs have limited use in TB diagnostics, they hold strong potential for early detection of LC and distinguishing LC from TB. These findings highlight the value of miRNAs as biomarkers in improving diagnostics for pulmonary diseases.

## Figures and Tables

**Table 1:** Demographic and clinical characteristics of the study groups in the exploratory and validation sets.

Characteristics	LC Patients		TB Patients		Healthy Controls	
	Profiling	Validation	Profiling	Validation	Profiling	Validation
Total	29	68	10	38	10	41
Male/female	25/4	47/21	8/2	17/21	8/2	30/11
Kazakhs/other ethnic groups *	25/4	56/12	8/2	34/4	10/0	39/2
Non-smokers/smokers	9/20	36/32	6/4	34/4	5/5	27/14
DS-TB/DR-TB	-	-	9/1	28/10	-	-
AC ^1^/SCC ^2^/SCLC ^3^/SA ^4^/AtC ^5^	10/10/9/0/0	32/25/8/2/1	-	-	-	-
Tumour stage (I/II/III/IV)	1/1/18/9	18/8/25/17	-	-	-	-
Age, years (mean ± SD)	62.0 ± 8.1	59. 7 ± 11.5	47.5 ± 13.3	36.5 ± 17.3	49.2 ± 6.5	58.5 ± 8.1

* Uighurs, Russians, Tatars, Mari and Dungans. ^1^ adenocarcinoma. ^2^ squamous cell carcinoma. ^3^ small cell carcinoma. ^4^ sarcoma. ^5^ atypical carcinoid.

**Table 2:** Comparative statistics of the levels of 14 miRNAs between the studied groups.

miRNA	LC Patients vs. TB Patients		LC Patients vs. Controls		TB Patients vs. Controls	
	Log_2_FC (95% CI)	*p*-Value	Log_2_FC (95% CI)	*p*-Value	Log_2_FC (95% CI)	*p*-Value
miR-130b-3p	2.30 (1.72; 2.86)	3.8 × 10^−11^ *	2.66 (2.09; 3.25)	2.6 × 10^−13^ *	0.34 (−0.19; 0.88)	0.188
miR-423-5p	1.80 (1.30; 2.35)	7.4 × 10^−10^ *	1.69 (1.23; 2.20)	1.5 × 10^−10^ *	−0.10 (−0.57; 0.38)	0.700
miR-15b-5p	4.31 (3.22; 5.26)	1.2 × 10^−9^ *	3.20 (2.19; 4.07)	1.3 × 10^−7^ *	−1.13 (−1.95; −0.29)	0.012 *
miR-18b-5p	2.05 (1.37; 2.83)	3.5 × 10^−7^ *	1.67 (1.00; 2.40)	2.2 × 10^−6^ *	−0.48 (−1.11; 0.28)	0.193
miR-1-3p	1.89 (1.19; 2.63)	7.4 × 10^−7^ *	2.29 (1.64; 2.95)	1.1 × 10^−10^ *	0.46 (−0.18; 1.04)	0.118
miR-200a-3p	1.24 (0.73; 1.71)	8.1 × 10^−6^ *	1.54 (1.09; 1.99)	7.1 × 10^−9^ *	0.31 (−0.14; 0.80)	0.167
miR-29b-2-5p	1.74 (0.57; 2.69)	0.0028 *	1.33 (0.37; 2.36)	0.0099 *	−0.46 (−1.40; 0.95)	0.517
miR-154-5p	1.56 (0.60; 2.44)	0.0029 *	1.06 (0.34; 1.88)	0.0045 *	−0.32 (−1.52; 0.99)	0.605
miR-375-3p	0.87 (0.33; 1.48)	0.0031 *	1.10 (0.53; 1.67)	5.7 × 10^−5^ *	0.16 (−0.32; 0.85)	0.555
miR-495-3p	1.77 (0.46; 3.07)	0.0081 *	0.90 (0.00; 1.89)	0.049	−0.78 (−2.17; 0.56)	0.255
miR-382-5p	1.47 (0.37; 2.64)	0.0084 *	1.12 (0.11; 2.10)	0.032	−0.57 (−1.41; 0.74)	0.523
miR-376a-3p	0.76 (0.17; 1.33)	0.010 *	1.57 (0.95; 2.26)	3.1 × 10^−6^ *	0.69 (0.14; 1.46)	0.021 *
miR-543	1.30 (0.14; 2.59)	0.036	0.69 (−0.28; 1.73)	0.166	−0.66 (−1.92; 0.70)	0.348
miR-204-5p	0.15 (−1.05; 1.29)	0.791	0.24 (−0.85; 1.31)	0.658	0.10 (−1.07; 1.20)	0.836

* remain significant after FDR correction for multiple comparisons.

## Data Availability

The raw data supporting the conclusions of this article will be made available by the authors upon request.

## Supplementary Materials

The following supporting information can be downloaded at: https://www.mdpi.com/article/10.3390/diagnostics14232684/s1, Table S1: Database; Table S2: Comparative statistics of the levels of 14 miRNAs between the studied groups in an independent validation sample set (after excluding samples from the exploratory stage); Table S3: Comparative statistics of the levels of 14 miRNAs among stage I LC patients, TB patients, and healthy controls; Table S4: Significant differences in miRNA levels between groups with different clinicopathological characteristics; Table S5: Results of regression analysis and partial correlation analysis to assess the impact of age on differences in miRNA levels between groups; Table S6: ROC-analysis results.
