# Supplementary material for: Circulating MicroRNAs as Biomarkers for the Early Diagnosis of Lung Cancer and Its Differentiation from Tuberculosis
Source: Diagnostics (Basel). 2024 Nov 28;14(23):2684. doi: 10.3390/diagnostics14232684 (PMC11640063; doi:10.3390/diagnostics14232684)
Supplement: Supplementary file 1 [file diagnostics-14-02684-s001.zip › Supplimentary Tables S2, S3, S4, S5, S6.pdf]

Table S2. Comparative statistics of the levels of 14 miRNAs between the studied groups in an independent validation sample set (after excluding samples from the exploratory stage)

**miRNA**	**LC patients vs. TB patients**		**LC patients vs. Controls**		**TB patients vs. Controls**	
	Log_2FC (95% CI)	*p*-Value	Log_2FC (95% CI)	*p*-Value	Log_2FC (95% CI)	*p*-Value
miR-130b-3p	2.31 (1.75; 2.89)	7.9×10^{-12}*	2.64 (2.05; 3.21)	8.9×10^{-13}*	0.30 (-0.18; 0.74)	0.207
miR-423-5p	1.89 (1.38; 2.44)	6.8×10^{-11}*	1.76 (1.29; 2.29)	1.4×10^{-10}*	-0.08 (-0.52; 0.33)	0.716
miR-15b-5p	4.24 (3.24; 5.24)	2.1×10^{-9}*	3.30 (2.35; 4.19)	2.6×10^{-8}*	-0.95 (-1.65; -0.31)	0.0065*
miR-18b-5p	2.03 (1.45; 2.66)	2.3×10^{-9}*	1.61 (1.01; 2.23)	4.0×10^{-7}*	-0.49 (-1.04; 0.13)	0.089
miR-200a-3p	1.48 (1.03; 1.89)	1.3×10^{-7}*	1.87 (1.43; 2.26)	2.1×10^{-11}*	0.35 (0.02; 0.76)	0.041
miR-1-3p	2.17 (1.34; 3.01)	7.4×10^{-7}*	2.34 (1.59; 3.20)	2.2×10^{-8}*	0.28 (-0.44; 0.92)	0.348
miR-154-5p	2.10 (1.30; 2.98)	4.3×10^{-6}*	0.98 (0.28; 1.86)	0.0074*	-1.06 (-2.03; 0.01)	0.057
miR-495-3p	2.39 (0.99; 3.67)	1.3×10^{-4}*	0.85 (-0.28; 2.04)	0.154	-1.51 (-2.87; -0.23)	0.024*
miR-375-3p	1.05 (0.52; 1.63)	1.4×10^{-4}*	1.34 (0.76; 1.90)	1.0×10^{-5}*	0.16 (-0.27; 0.74)	0.538
miR-382-5p	1.95 (0.97; 3.09)	2.3×10^{-4}*	1.23 (0.27; 2.30)	0.016*	-0.86 (-1.61; 0.33)	0.166
miR-29b-2-5p	1.74 (0.56; 2.69)	0.0029*	1.48 (0.50; 2.61)	0.0051*	-0.22 (-1.36; 1.26)	0.789
miR-543	1.82 (0.74; 3.25)	0.0034*	0.39 (-0.60; 1.51)	0.397	-1.47 (-3.04; -0.28)	0.019
miR-376a-3p	0.79 (0.17; 1.39)	0.014*	1.64 (0.94; 2.45)	4.2×10^{-5}*	0.78 (0.12; 1.57)	0.026*
miR-204-5p	0.51 (-0.63; 1.61)	0.361	0.47 (-0.60; 1.49)	0.401	-0.01 (-1.05; 0.88)	0.979

* remain significant after FDR correction for multiple comparisons

Table S3. Comparative statistics of the levels of 14 miRNAs among stage I LC patients, TB patients, and healthy contorls

miRNA	st.I LC patients vs. TB patients		LC patients vs. Controls	
	Log_2FC (95% CI)	*p*-Value	Log_2FC (95% CI)	*p*-Value
miR-15b-5p	4.66 (3.23; 6.22)	9.3×10^{-8}*	3.45 (2.03; 4.99)	5.6×10^{-6}*
miR-130b-3p	2.49 (1.49; 3.46)	2.5×10^{-7}*	2.83 (1.80; 3.80)	1.2×10^{-8}*
miR-423-5p	1.96 (1.09; 3.10)	1.3×10^{-5}*	1.92 (0.98; 2.91)	1.7×10^{-5}*
miR-18b-5p	2.38 (1.36; 3.44)	2.2×10^{-5}*	1.87 (0.93; 2.96)	9.7×10^{-5}*
miR-200a-3p	1.05 (0.45; 1.74)	0.0010*	1.32 (0.76; 1.98)	9.5×10^{-5}*
miR-1-3p	1.98 (0.70; 3.07)	0.0011*	2.12 (1.04; 3.57)	1.3×10^{-5}*
miR-154-5p	2.08 (0.68; 3.26)	0.0037*	1.52 (0.55; 2.67)	0.0013*
miR-495-3p	2.44 (0.52; 4.32)	0.012*	1.50 (0.17; 3.12)	0.034*
miR-382-5p	1.91 (0.31; 3.53)	0.018*	1.48 (0.02; 2.77)	0.046
miR-376a-3p	0.99 (0.16; 1.84)	0.022*	1.75 (0.92; 2.78)	1.5×10^{-4}*
miR-543	2.19 (0.28; 3.75)	0.024*	1.50 (-0.34; 2.71)	0.118
miR-29b-2-5p	1.69 (0.10; 2.86)	0.038*	1.35 (-0.25; 2.79)	0.072
miR-375-3p	0.62 (0.00; 1.22)	0.053	0.77 (0.16; 1.44)	0.011*
miR-204-5p	-0.98 (-2.88; 0.81)	0.238	-0.92 (-2.65; 0.61)	0.236

* remain significant after FDR correction for multiple comparisons

Table S4. Significant differences in miRNA levels between groups with different clinicopathological characteristics

Compared groups	miRNA	Log_2FC (95% CI)	*p*-Value
AC patients vs. SCC patients	miR-200a-3p	0.67 (0.16; 1.32)	0.019
	miR-375-3p	0.83 (0.11; 1.58)	0.020
SCLC patients vs. AC patients	miR-18b-5p	2.33 (0.88; 3.79)	0.0033
	miR-154-5p	1.37 (0.34; 2.30)	0.0076
	miR-375-3p	2.02 (0.30; 4.42)	0.022
	miR-29b-2-5p	1.93 (0.15; 3.85)	0.039
SCLC patients vs. SCC patients	miR-18b-5p	2.99 (1.50; 4.61)	5.8×10^{-4} *
	miR-375-3p	3.09 (1.23; 5.28)	0.0011 *
	miR-154-5p	2.02 (0.64; 3.91)	0.0094
	miR-130b-3p	1.73 (0.27; 3.30)	0.020
	miR-200a-3p	1.19 (0.09; 2.74)	0.036
	miR-495-3p	1.91 (0.04; 4.08)	0.041
SCLC patients vs. NSCLC patients	miR-18b-5p	2.62 (1.27; 3.94)	0.0014 *
	miR-375-3p	2.7 (0.82; 4.69)	0.0050
	miR-154-5p	1.57 (0.56; 2.72)	0.0057
	miR-130b-3p	1.52 (0.18; 2.87)	0.026
	miR-29b-2-5p	1.9 (0.16; 3.78)	0.034
	miR-495-3p	1.75 (0.12; 3.73)	0.036
	miR-15b-5p	2.26 (0.01; 4.48)	0.046
Metastases in LC patients: yes vs. no	miR-375-3p	1.04 (0.24; 2.01)	0.014
TB patients: smokers vs. non-smokers	miR-204-5p	2.58 (0.34; 5.47)	0.042
Controls: smokers vs. non-smokers	miR-423-5p	0.66 (0.01; 1.32)	0.048

* remain significant after FDR correction for multiple comparisons

Table S5. Results of regression analysis and partial correlation analysis to assess the impact of age on differences in miRNA levels between groups

miRNA	LC patients vs. TB patients			TB patients vs. Controls		
	**Linear regression, *p*-Value**		**Partial correlation, *p*-Value**	**Linear regression, *p*-Value**		**Partial correlation, *p*-Value**
	Factor: groups	Covariate: age		Factor: groups	Covariate: age	
miR-130b-3p	1.4×10^{-6}	0.414	5.6×10^{-9}	0.559	0.682	0.282
miR-423-5p	7.7×10^{-6}	0.465	3.4×10^{-9}	0.929	0.754	0.406
miR-15b-5p	2.3×10^{-6}	0.976	9.0×10^{-9}	0.0069	0.123	0.0016
miR-18b-5p	2.6×10^{-4}	0.600	9.6×10^{-6}	0.320	0.646	0.073
miR-1-3p	9.0×10^{-4}	0.138	1.2×10^{-4}	0.458	0.538	0.580
miR-200a-3p	0.011	0.150	5.6×10^{-4}	0.365	0.684	0.771
miR-29b-2-5p	0.030	0.748	0.0099	0.100	0.039***	0.031**
miR-154-5p	0.069*	0.210	0.0045	0.301	0.046***	0.472
miR-375-3p	0.014	0.661	5.7×10^{-5}	0.958	0.135	0.413
miR-495-3p	0.014	0.911	0.049	0.114	0.250	0.123
miR-382-5p	0.146*	0.465	0.032	0.918	0.461	0.663
miR-376a-3p	0.0072	0.463	3.1×10^{-6}	0.668*	0.097	0.240*
miR-543	0.572*	0.174	0.166*	0.708	0.650	0.718
miR-204-5p	0.597	0.382	0.658	0.802	0.848	0.932

* differences became non-significant; ** differences became significant; *** time has a significant effect

Table S6. ROC-analysis results

Classes	Potential markers	AUC (95% CI)	Optimal cut-of-value	Sensitivity (95% CI)	Specificity (95% CI)
LC patients vs. Controls	miR-130b-3p	0.920 (0.871–0.973)	-7.70	0.882 (0.806-0.959)	0.829 (0.714-0.944)
LC patients vs. Controls	miR-1-3p	0.870 (0.810–0.932)	-6.65	0.735 (0.630–0.840)	0.878 (0.778–0.978)
LC patients vs. Controls	miR-423-5p	0.867 (0.802–0.938)	-3.90	0.691(0.581–0.801)	0.902 (0.812–0.993)
LC patients vs. Controls	miR-200a-3p	0.832 (0.757–0.909)	-10.74	0.926 (0.864–0.989)	0.610 (0.460–0.759)
LC patients vs. Controls	miR-15b-5p	0.803 (0.714–0.894)	-4.94	0.776 (0.676–0.876)	0.805 (0.684–0.926)
LC patients vs. Controls	miR-18b-5p	0.774 (0.684–0.870)	-9.50	0.853 (0.769–0.937)	0.625 (0.475–0.775)
LC patients vs. Controls	miR-376a-3p	0.768 (0.683–0.855)	-9.05	0.529 (0.411–0.648)	0.878 (0.778–0.978)
LC patients vs. Controls	miR-375-3p	0.731 (0.640–0.828)	-4.83	0.794 (0.698–0.890)	0.634 (0.487–0.782)
LC patients vs. Controls	miR-154-5p	0.694 (0.573–0.811)	-12.05	0.567 (0.441–0.692)	0.769 (0.607–0.931)
LC patients vs. Controls	miR-29b-2-5p	0.658 (0.546–0.767)	-11.61	0.578 (0.457-0.699)	0.743 (0.598-0.888)
LC patients vs. Controls	miR-382-5p	0.633 (0.514–0.746)	-11.75	0.435 (0.312–0.559)	0.824 (0.695–0.952)
LC patients vs. Controls	miR-495-3p	0.622 (0.506–0.739)	-12.47	0.683(0.568–0.797)	0.529 (0.362–0.697)
TB patients vs. Controls	miR-15b-5p	0.666 (0.544-0.789)	-6.77	0.659 (0.513-0.804)	0.649 (0.495-0.802)
TB patients vs. Controls	miR-376a-3p	0.650 (0.532-0.775)	-10.43	0.789 (0.660-0.919)	0.561 (0.409-0.713)
LC patients vs. TB patients	miR-130b-3p	0.889 (0.822–0.954)	-7.80	0.897 (0.825-0.969)	0.763 (0.628-0.898)
LC patients vs. TB patients	miR-423-5p	0.862 (0.789–0.936)	-4.50	0.853 (0.769–0.937)	0.763 (0.628–0.898)
LC patients vs. TB patients	miR-15b-5p	0.862 (0.785–0.937)	-5.46	0.866 (0.784–0.947)	0.838 (0.719–0.957)
LC patients vs. TB patients	miR-18b-5p	0.800 (0.704–0.898)	-9.50	0.853 (0.769–0.937)	0.711 (0.566–0.855)
LC patients vs. TB patients	miR-1-3p	0.791 (0.706–0.875)	-4.65	0.735 (0.630–0.840)	0.737 (0.597–0.877)
LC patients vs. TB patients	miR-200a-3p	0.762 (0.661-0.869)	-10.29	0.809 (0.715-0.902)	0.711 (0.566-0.855)
LC patients vs. TB patients	miR-154-5p	0.703 (0.565-0.838)	-13.15	0.833 (0.739-0.928)	0.577 (0.387-0.767)
LC patients vs. TB patients	miR-29b-2-5p	0.692 (0.576-0.810)	-12.173	0.641 (0.523-0.758)	0.733 (0.575-0.892)
LC patients vs. TB patients	miR-495-3p	0.682 (0.551-0.814)	-12.03	0.556 (0.433-0.678)	0.760 (0.593-0.927)
LC patients vs. TB patients	miR-382-5p	0.675 (0.556-0.797)	-13.442	0.726 (0.615-0.837)	0.679 (0.506-0.852)
LC patients vs. TB patients	miR-375-3p	0.674 (0.567-0.786)	-4.90	0.824 (0.733-0.914)	0.579 (0.422-0.736)
LC patients vs. TB patients	miR-376a-3p	0.651 (0.546-0.757)	-8.92	0.471 (0.352-0.589)	0.842 (0.726-0.958)
LC patients vs. TB patients	miR-543	0.647 (0.515-0.777)	-13.26	0.800 (0.699-0.901)	0.500 (0.300-0.700)
SCLC patients vs. NSCLC patients	miR-18b-5p	0.853 (0.739-0.966)	-7.27	0.875 (0.646-1.000)	0.789 (0.684-0.895)
SCLC patients vs. NSCLC patients	miR-375-3p	0.809 (0.629-0.993)	-3.48	0.875 (0.646-1.000)	0.684 (0.564-0.805)
SCLC patients vs. NSCLC patients	miR-154-5p	0.807 (0.675-0.935)	-11.50	0.875 (0.646-1.000)	0.700 (0.573-0.827)
SCLC patients vs. NSCLC patients	miR-130b-3p	0.746 (0.582-0.912)	-6.42	1.000 (1.000-1.000)	0.474 (0.344-0.603)
SCLC patients vs. NSCLC patients	miR-29b-2-5p	0.736 (0.573-0.900)	-12.51	1.000 (1.000-1.000)	0.415 (0.282-0.548)
SCLC patients vs. NSCLC patients	miR-495-3p	0.733 (0.553-0.917)	-10.86	0.750 (0.450-1.000)	0.731 (0.610-0.851)
SCLC patients vs. NSCLC patients	miR-15b-5p	0.721 (0.538-0.906)	-2.84	0.875 (0.646-1.000)	0.518 (0.387-0.649)
AC patients vs. SCC patients	miR-200a-3p	0.683 (0.540-0.823)	-8.92	0.500 (0.327-0.673)	0.880 (0.753-1.000)
AC patients vs. SCC patients	miR-375-3p	0.682 (0.534-0.829)	-4.64	0.750 (0.600-0.900)	0.560 (0.365-0.755)
Metastases in LC patients: Yes vs. no	miR-375-3p	0.711 (0.565–0.860)	-4.42	0.875 (0.713–1.000)	0.481 (0.345–0.617)
